# Selenium Supplementation May Decrease Thyroid Peroxidase Antibody Titer via Reducing Oxidative Stress in Euthyroid Patients with Autoimmune Thyroiditis

**DOI:** 10.1155/2020/9210572

**Published:** 2020-06-30

**Authors:** Xun Tian, Ning Li, Rui Su, Chenyang Dai, Ruiguo Zhang

**Affiliations:** ^1^Tianjin Institute of Hepatology, Tianjin Second People's Hospital, Tianjin, China; ^2^Department of Nuclear Medicine, Tianjin Medical University General Hospital, Tianjin, China

## Abstract

**Objective:**

Selenium, as an antioxidant, has been implicated in the development of autoimmune thyroiditis (AIT). Many studies showed selenium supplementation could decrease thyroid autoantibodies in patients with AIT. However, the underlying mechanisms have not been well determined. Therefore, we performed a clinical study to investigate the possible mechanism of beneficial effects of selenium treatment on AIT patients.

**Methods:**

Forty euthyroid patients with AIT were randomized into two groups. Group I was treated with 200 *μ*g/day selenium supplementation, and group II received a placebo over a 3-month period. Thyroid stimulating hormone (TSH), thyroid peroxidase antibody (TPOAb), antithyroglobulin antibody (TgAb), malondialdehyde (MDA), total antioxidant capacity (TAC), and superoxide dismutase (SOD) were measured before and 3 months after treatments. Additionally, twenty healthy volunteers also served as a control group for the evaluation of such parameters in basic condition.

**Results:**

Totally, 32 patients (group I, *n* = 18; group II, *n* = 14) completed the clinical study and were incorporated into the statistics. MDA level was higher and SOD activity and TAC were lower in patients compared to healthy individuals. After 3 months, TPOAb titer significantly decreased within group I (*P* < 0.001) but did not change within group II (*P*=0.001). There were also no statistically significant changes in TSH and TgAb titers within the two groups (all *P* > 0.05). Additionally, decreased MDA level (from 6.8 ± 1.3 nmol/ml to 4.9 ± 0.7 nmol/ml; *P* < 0.001) and increased TAC (from 10.0 ± 1.9 mmol/l to 12.9 ± 3.1 mmol/l; *P*=0.003) and SOD activity (from 72.3 ± 10.3 U/ml to 84.3 ± 13.2 U/ml; *P*=0.007) were simultaneously observed after 3 months' selenium treatment. Moreover, there was a negative correlation between TAC and TgAb/TPOAb and a positive correlation between MDA and TgAb/TPOAb in AIT patients.

**Conclusions:**

Our findings support the hypothesis that selenium treatment could decrease TPOAb titer via enforcing the defense against oxidative stress in euthyroid patients with AIT, which may be a potential underlying mechanism.

## 1. Introduction

Autoimmune thyroiditis (AIT) is a chronic autoimmune inflammatory disease of the thyroid gland. The T and B lymphocytes contribute to the pathogenesis of AIT by the production of autoantibodies to thyroglobulin and thyroid peroxidase and excessive reactive oxygen species (ROS) under the environmental influence or thyroid dysfunction [1]. Oxidative stress is defined as an imbalance between the production of ROS and antioxidant defenses. Excessive oxidative stress could cause damage to human proteins, lipids, and nucleic acids [2]. At present, the biomarkers of oxidative stress, such as malondialdehyde (MDA), total antioxidant capacity (TAC), total oxidant status (TOS), and superoxide dismutase (SOD), are often used to evaluate the oxidative and antioxidative status in vivo, because there has been no single method that can accurately measure ROS [2]. Several studies have shown increased oxidative status and decreased antioxidant defense capacity in AIT patients compared to healthy individuals [1, 3–6], whereas most of them do not focus on or ignore the impacts of thyroid dysfunction on the oxidative stress.

Selenium, as an essential trace element, is also served as an antioxidant, and selenium deficiency has been proven to be associated with many diseases, such as cancer, diabetes, and AIT [5, 7–9]. Wu et al. reported that the incidence of AIT is higher in the areas with selenium deficiency, but the exact mechanism remains unclear [10]. Recently, excessive oxidative stress has been implicated in the pathogenesis of AIT, and some authors support the opinion of selenium deficiency in the development of AIT and the potentially beneficial effects of selenium supplementation on AIT [11–14]. However, the underlying mechanisms of slowing down and preventing the development of AIT have not been fully clarified, and some studies even contrastly showed the different even opposite results. In a meta-analysis, Wichman et al. recommended clinicians not to warrant routine use of selenium supplementation in the treatment of patients with AIT while correction of selenium deficiency seems rational in order to avoid negative health effects [15]. Several studies revealed that selenium supplementation had no significant effects on the decrease of thyroid autoantibodies titers and thus yielded conflicting results [16, 17]. Therefore, further studies are needed to evaluate the benefits of selenium supplementation on AIT patients.

There is growing evidence that oxidative stress is closely correlated with thyroid function. Previous studies have indicated an imbalance between oxidant stress and the defense system in hyperthyroidism [18–20]. Additionally, induction of euthyroidism after thiamazole therapy for patients with Graves' diseases results in the resolution of unbalanced oxidant/antioxidant status [21]. Similarly, oxidative stress increased and antioxidant defense capacity decreased in hypothyroid patients, and 6 months of levothyroxine replacement could decrease oxidant status and increases antioxidant capacity [22, 23]. Therefore, it is important to take into account the effect of thyroid function when we evaluate oxidative stress and it is interesting to investigate the correlation between thyroid autoantibodies and oxidative stress indexes in euthyroid patients with newly diagnosed AIT and further to determine the impact of early selenium supplementation on delaying or preventing the progression of AIT.

The present study was to evaluate the oxidant/antioxidant status in euthyroid patients with AIT and determine the correlation between oxidative stress parameters (MDA, TAC, and SOD) and thyroid autoantibodies. Furthermore, we investigated whether selenium supplementation could decrease thyroid autoantibodies and reduce autoimmune responses by scavenging free radicals in newly diagnosed AIT patients.

## 2. Materials and Methods

### 2.1. Patient Selection

This is a prospective placebo-controlled randomized study carried out in our hospital between September 2016 and August 2017. A total of 40 patients with euthyroidism, newly diagnosed with AIT, and aged over 18 years old (mean age, 40.2 ± 10.9 years) were enrolled in this study. AIT was diagnosed when the serum thyroid peroxidase antibody (TPOAb) and/or thyroglobulin antibody (TgAb) was positive together with the presence of parenchymal heterogeneity in high-resolution sonography.

Patients who had nonthyroidal disorders including cancer, hypertension, diabetes mellitus, coronary artery disease, chronic kidney disease, liver diseases, heart failure, cerebrovascular disease and rheumatism, smokers, use of other antioxidant agents or vitamin supplements within the past 6 months and those who were pregnant were not included in this study. All patients were randomized into two groups. Group I received 200 *μ*g/day selenium supplementation (selenious yeast tablets, Lingtai Pharmaceutical Co., Ltd. Mudanjiang, China) orally for 3 months, and group II received placebo.

Moreover, age, sex, and body mass index (BMI) matched 20 healthy volunteers were served as a control group for the evaluation of oxidative stress parameters in basic condition. This study is conducted in accordance with the Declaration of Helsinki and was approved by the Ethics Research Committee of Tianjin Second People's Hospital, and informed consent was obtained from each patient and control subject.

### 2.2. Sample Collection

Venous blood samples were obtained after overnight fasting at pretreatment (baseline) for patients and healthy volunteers and at 3 months posttreatment for patients, and measured in plasma samples (collected in tubes containing 16 IU/ml heparin) for oxidative stress parameters and in serum samples for thyroid function and autoantibodies. After centrifugation at 4000 rpm for 10 minutes, the collected samples were stored at −80°C until assay.

### 2.3. Measurement of Biochemical Markers

Serum free triiodothyronine (FT_3_, reference 3.50–6.50 pmol/L), free thyroxine (FT_4_, reference 11.50–23.50 pmol/L) and TSH (reference 0.30–5.00 *μ*IU/mL) levels, and TgAb (reference 0–40.00 IU/mL) and TPOAb (reference 0–35.00 IU/mL) titers were measured by a fully automated ADVIA Centaur analyzer (Siemens Healthcare Diagnostics, New York, USA) based on chemiluminescent reaction principle. Selenium levels were determined by absorption spectroscopy (Biosyn, Feldbach, Germany).

### 2.4. Measurement of Oxidative Stress Markers

#### 2.4.1. Measurement of MDA

MDA level (nmol/ml) was estimated with the method based on the reaction of MDA with thiobarbituric acid (TBA) to form thiobarbituric acid-reactive substances (TBARS) [24]. Briefly, 2.4 mL of trichloroacetic acid was mixed with 0.3 mL of serum, and 0.3 mL of 10% phosphotungstic acid was added. Then, the mixture was centrifuged at 1,600 ×g for 10 minutes. The supernatant was discarded, and the sediment was suspended in 4 mL of distilled water. Subsequently, 1 mL of 0.67% TBA was added, and the mixture was heated at 95°C for 60 minutes. The formed color was extracted into *n*-butanol. The mixture was centrifuged at 1600 ×g for 10 minutes. The absorbance of the resultant supernatant was read at 532 nm.

#### 2.4.2. Measurement of SOD

Superoxide dismutase (SOD) activity (U/ml) was measured using the method described previously [25], which consists of the inhibition of nitroblue tetrazolium (NBT) reduction with xanthine-xanthine oxidase used as a superoxide generator. The activity of SOD was determined by measuring the reduction in optical density of the reaction solution at 550 nm with a spectrophotometer.

#### 2.4.3. Measurement of TAC

TAC was measured using a commercial kit based on the ability of antioxidants in plasma to inhibit the oxidation of 2,2′-azino-di(3-ethylbenzthiazoline sulphonate) (ABTS) to ABTS + by metmyoglobin [26]. The absorbance is measured at 750 nm. The capacity of the antioxidants in plasma to prevent ABTS oxidation is compared with that of Trolox, a water-soluble tocopherol analogue, and the results were expressed as millimoles Trolox equivalent/liter (mmol/l).

#### 2.4.4. Statistical Analysis

All data were analyzed by SPSS software (version 18.0, SPSS Inc, Chicago, IL). The normal distribution of data was evaluated with the Kolmogorov–Smirnov test. Numerical variables were expressed as mean ± standard deviation (SD) or median (25th–75th percentile) depending on normal distribution or not. A Student's *t*-test or Mann–Whitney *U* test was used to compare the baseline parameters between the AIT patients and healthy controls. A Chi-square test was used to compare the categorical data. Changes in the variables pre- and posttreatment were assessed using a paired samples *t*-test or Wilcoxon signed rank test. Correlation analyses were performed with a Pearson's correlation test. A *P* < 0.05 was considered statistically significant.

## 3. Results

### 3.1. Patients Characteristics and the Comparison between Patients and Controls

The population of this study consisted of 40 patients with AIT and 20 healthy controls. During the study, 4 patients in the selenium supplementation group (group I) and 4 in the placebo group (group II) dropped out of this research because of not insisting on taking drugs. Finally, 32 patients completed this study. No significant adverse events were reported by the participants.

The basal characteristics of patients and healthy controls are presented in Table 1. There were no statistically significant differences between AIT patients and controls in age, sex, FT_3_, FT_4_, TSH, and BMI (all *P* > 0.05). Although selenium levels in the patient group were lower than those in the control group, they were both in the normal range.

The plasma oxidative stress parameters of patients and controls are shown in Table 2. The mean MDA level was higher in AIT patients in comparison to the healthy controls (6.9 ± 1.4 vs. 4.1 ± 0.9, *P*=0.004) whereas the mean TAC and SOD activities were lower in patients than the controls (both *P* < 0.001).

### 3.2. Changes of Indexes before and after Treatment within the Two Groups

**Figure 1:**
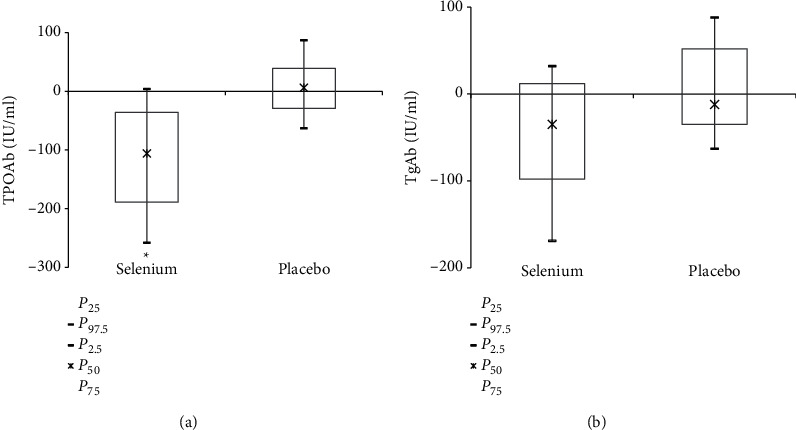
Comparison of the changes (Δ) of thyroid autoantibodies titers within the two groups. A Wilcoxon signed-rank test was used to determine the changes from post- to pretreatment (post-pre). (a) Serum TPOAb titers (IU/ml). (b) Serum TgAb titers (IU/ml). The data were shown as median (*P*_25_, *P*_75_). ^*∗*^*P* < 0.05.

The changes of oxidative stress parameters and thyroid autoantibodies before and after 3 months of selenium (group I, *n* = 18) or placebo (group II, *n* = 14) treatment are shown in Table 3. All patients' levels of TSH and FT_4_ were still normal after treatment. With regard to the thyroid autoantibodies, after selenium supplementation, TPOAb titer significantly decreased at 3 months compared to baseline (post-pre, −106.0 (−36, −189), *P* < 0.001) whereas there was no obvious change in the placebo group (post-pre, 6.0(39, −29), *P*=0.110) (Figure 1(a)). Moreover, no statistically significant differences were found regarding their TgAb titer within group I (post-pre, −35.0 (12, −98), *P*=0.081) and group II (post-pre, −12 (52, −35), *P*=0.363) (Figure 1(b)).

**Figure 2:**
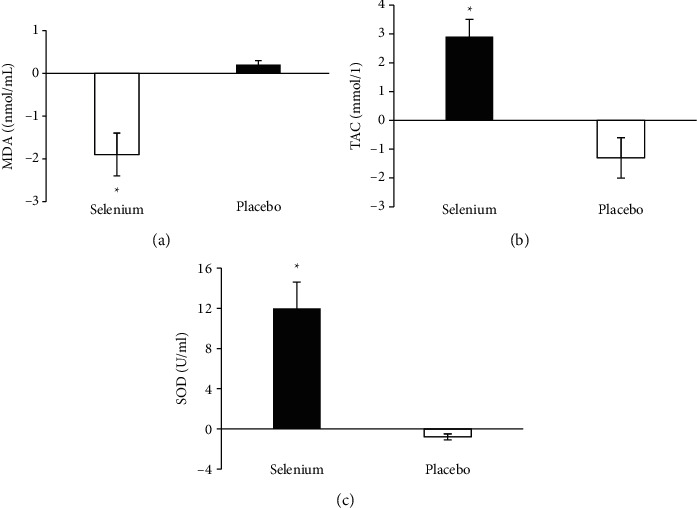
Comparison of the changes (Δ) of oxidative stress markers within the two groups. A paired-samples *t*-test was used to determine the changes from post- to pretreatment (post-pre). (a) Plasma MDA levels (nmol/ml). (b) Plasma TAC activity (mmol/l). (c) Plasma SOD activity (U/ml). The data were shown as standard deviation. ^*∗*^*P* < 0.05.

Similarly, regarding the oxidative stress indexes, after selenium treatment, a paired sample *t*-test indicated a significant decrease in MDA level (post-pre, −1.9 ± 0.5 nmol/ml, *P* < 0.001) and increase in TAC (2.9 ± 0.6 mmol/l, *P*=0.003) and SOD (12.0 ± 2.6 U/ml, *P*=0.007) activity in the selenium group, whereas there were no significant changes for the three indexes in the placebo group (all *P* > 0.05) (Figures 2(a)–2(c)).

### 3.3. Correlation between Oxidative Stress Parameters and Thyroid Autoantibodies Titers

At baseline, the correlation analysis between oxidative stress parameters and thyroid autoantibodies titers is demonstrated in Table 4. There were negative correlations between TAC and TgAb or TPOAb (*r* = −0.268, *P*=0.039 and *r* = −0.463, *P*=0.008; respectively) and positive correlations between MDA and TgAb or TPOAb (*r* = 0.429, *P*=0.041 and *r* = 0.587, *P*=0.023; respectively) in the AIT patients, but no obvious relationships were found between them in the healthy controls. Additionally, we did not find any correlation between SOD and the two thyroid autoantibodies in the two groups.

## 4. Discussion

In this prospective study, we demonstrated that the MDA level was higher, while antioxidative defense capacity was lower in euthyroid patients with AIT as compared to healthy individuals. Selenium supplementation could not only reduce the oxidative stress, but to some extent, decrease the TPOAb titer. Additionally, we found that TPOAb and TgAb correlated positively with MDA and negatively with TAC, suggesting an interdependent relationship between thyroid autoimmunity and enhanced oxidative stress in AIT patients. We suspect that selenium treatment may decrease TPOAb titer through reducing oxidative stress, and this can be served as a potential underlying mechanism of inhibiting thyroid autoimmune response in the development of AIT.

Oxidative stress occurs as a result of either overproduction of ROS or insufficiency of antioxidant defense systems [2]. Generally, mild to moderate oxidative stress is essential for maintaining redox homeostasis and regulating life processes as well as for enhancing the expression of antioxidant enzymes. Contrarily, excessive oxidative stress is responsible for damaging biomolecules and disrupting redox signaling, and is, therefore, implicated in the pathogenesis of the major human diseases [7, 18, 27, 28]. ROS are difficult to measure as they are metabolized rapidly in vivo. MDA, a strong oxidant, is the end product of lipid peroxidation and commonly used as a biomarker to evaluate the oxidative stress level. TAC reflects the whole oxygen radical absorbance capacity and general antioxidative status in the body and is the most reliable factor involved in antioxidation protection. SOD, as one of the main antioxidative enzymes, is considered as the first step of defense system against ROS.

AIT is a chronic autoimmune disease of the thyroid gland. Physiological ROS is essential for the synthesis of thyroid hormone [18]. However, the inflammation in AIT can result from the T and B lymphocytes activation, which in a similar way causes excessive ROS through nicotinamide adenine dinucleotide phosphate oxidase enzyme when they are stimulated and further leads to an increase in thyroid autoantibodies titers [5]. In the present study, MDA was significantly higher, while TAC and SOD activities were significantly lower in patients with AIT compared with the healthy controls, which were consistent with the previous research studies [1, 4, 5, 29], indicating increased oxidative stress in AIT patients. Our study revealed that oxidative stress played a role in the pathogenesis and progression of AIT. We consider that overproduction of ROS in AIT patients leads to excessive consumption of antioxidants, which results in a decrease in antioxidant activity.

Selenium, as a trace element, is essential for life. Various diseases, such as cancer, diabetes, and AIT, have been proven to be associated with selenium deficiency [5, 8, 9]. Additionally, the thyroid gland is one of the organs with the highest selenium concentration, and selenium, like iodine, is essential for normal thyroid function and thyroid hormone homeostasis. Contrarily, selenium deficiency, by impairing deiodinases activity, decreases the synthesis of triiodothyronine (T3) [18, 30, 31]. At present, more and more studies have shown a beneficial effect of selenium supplementation on AIT and other autoimmune disorders such as asthma and rheumatoid arthritis by the improvement of autoimmune response [4, 5, 12, 14, 15, 32–34]. A study conducted by Mantovani et al. showed oral selenium supplementation had an effect in reducing TPOAb concentrations and the recurrence of postpartum thyroiditis during pregnancy [11]. In a recent meta-analysis, Wichman et al. reported that selenium supplementation could decrease serum TPOAb titer after 3, 6, and 12 months and TgAb titer at 12 months in an LT_4_-treated AIT population, while reduce TPOAb and TgAb titers only at 3 months in an untreated AIT population [15]. The present study also showed significantly decreased TPOAb titer after 3 months of selenium supplementation in patients with AIT, while revealed no significant changes for TgAb titer. However, Karanikas et al. found no additional benefits or significant change in TPOAb titer after 3 months of selenium treatment [17]. This was in agreement with the previous study [35]. There are several reasons for explaining the differences. Firstly, Tg, in contrast to TPO, is not necessarily an antigen only expressed during a thyroid-specific autoimmune response. Therefore, TgAb is less specific than TPOAb for AIT. Additionally, the different baseline titers of TPOAb and TgAb before selenium supplementation and diverse thyroid function status (e.g., subclinical hypothyroidism or overt hypothyroidism, with or without levothyroxine treatment), to some extent, may contribute to the inconsistent results.

Growing evidence showed increased oxidative stress was not only closely correlated with thyroid dysfunction, including hypothyroidism and hyperthyroidism, but had significant influences on the pathogenesis of AIT [20, 23]. Previous studies demonstrated TAC correlated negatively with anti-TG and anti-TPO while TOS positively with anti-TG in euthyroid patients with AIT and HT patients developing overt hypothyroidism [5, 29], which were consistent with our study. However, no correlation was found between TOS and anti-TPO in the study by Baser et al. [29] and Ates et al. even reported TAC negatively correlated with anti-TG only in subclinical hypothyroid patients with HT, but not in euthyroid and hypothyroid cases with HT [1]. We speculate the differences could be attributed to the different thyroid function status and the degree of AIT. Additionally, the different methods for determining oxidative stress indexes may also contribute to the inconsistent results shown by different authors. Besides, our study investigated the relationship between SOD activity and thyroid autoantibodies in AIT, and interestingly found no correlation between them, indicating that the degree reduction of SOD is not proportional to the decrease of TPOAb or TgAb, though SOD activity is lower in AIT patients as compared to healthy individuals.

There are certain limitations to our study. Our sample size was small, and we only used MDA, TAC, and SOD for the evaluation of oxidative stress because the methods were established to be easy, rapid, reliable, and inexpensive, while we did not measure oxidative stress index (OSI), 8-iso-PGF2a or 8-hydroxy-2′-deoxyguanosine levels which are widely used as biomarkers of oxidative damage.

In conclusion, our study demonstrates an imbalance between oxidant stress and the defense system in patients with euthyroid AIT. The correlation between thyroid antibodies and biomarkers of oxidative stress may reflect the role of oxidative stress in the development of autoimmunity. Additionally, selenium supplementation could decrease TPOAb titer, which may play a role via enforcing the defense against oxidative stress in euthyroid patients with AIT.

## Figures and Tables

**Table 1:** Demographic and clinical data in patients with autoimmune thyroiditis and healthy controls.

Variables	Patients (*n* = 32)	Controls (*n* = 20)	*P*
Age (years)	41.6 ± 6.8	42.3 ± 5.4	0.699
Sex (F/M)	20/12	12/8	0.857
FT_3_ (pmol/L)	4.3 ± 0.8	4.6 ± 0.5	0.140
FT_4_ (pmol/L)	15.0 ± 3.2	14.2 ± 3.5	0.402
TSH (µIU/mL)	2.8 (0.9–4.2)	1.9 (0.7–4.0)	0.231
TPOAb (IU/mL)	587.0 (99.0–812.0)	23.0 (12.0–28.0)	<0.001
TgAb (IU/mL)	489.0 (38.0–702.0)	28.0 (20.0–32.0)	<0.001
BMI(kg/m^2^)	24.1 ± 4.1	23.6 ± 3.2	0.645
Selenium (*μ*g/L)	109.8 ± 16.3	123.1 ± 19.1	0.010

FT_3_: free triiodothyronine; FT_4_: free thyroxine; TSH: thyroid stimulating hormone; TPOAb: thyroid peroxidase antibody; TgAb: antithyroglobulin antibody; BMI: body mass index.

**Table 2:** Oxidative status markers in patients with autoimmune thyroiditis and healthy controls.

Variables	Patients (*n* = 32)	Controls (*n* = 20)	*P*
MDA (nmol/ml)	6.9 ± 1.4	4.1 ± 0.9	0.004
TAC (mmol/l)	10.2 ± 2.2	14.1 ± 3.8	<0.001
SOD (U/ml)	70.9 ± 9.5	95.1 ± 16.4	<0.001

MDA: malondialdehyde; TAC: total antioxidant capacity; SOD: superoxide dismutase.

**Table 3:** Comparison of thyroid autoantibodies and oxidative stress markers before and after treatment within each group.

Group	Pretreatment	Posttreatment	*P*
TPOAb (IU/ml)			
Selenium (*n* = 18)	603.0 (121.0, 846.0)	497.0 (82.0, 706.0)	<0.001
Placebo (*n* = 14)	581.0 (91.0, 802.0)	569.0 (90.0, 814.0)	0.110

TgAb (IU/ml)			
Selenium (*n* = 18)	482.0 (92.0, 718.0)	454.0 (89.0, 716.0)	0.081
Placebo (*n* = 14)	501.0 (46.0, 692.0)	486.0 (50.0, 702.0)	0.363

TSH (*μ*IU/mL)			
Selenium (*n* = 18)	1.69 (1.10, 3.02)	1.41 (0.58, 2.17)	0.734
Placebo (*n* = 14)	1.94 (0.92, 2.77)	2.12 (1.78, 3.56)	0.935

MDA (nmol/ml)			
Selenium (*n* = 18)	6.8 ± 1.3	4.9 ± 0.7	<0.001
Placebo (*n* = 14)	7.0 ± 1.6	7.2 ± 1.2	0.700

TAC (mmol/l)			
Selenium (*n* = 18)	10.0 ± 1.9	12.9 ± 3.1	0.003
Placebo (*n* = 14)	10.5 ± 2.5	9.2 ± 2.7	0.171

SOD (U/ml)			
Selenium (*n* = 18)	72.3 ± 10.3	84.3 ± 13.2	0.007
Placebo (*n* = 14)	69.1 ± 9.1	68.3 ± 11.4	0.832

TSH: thyroid stimulating hormone; TPOAb: thyroid peroxidase antibody; TgAb: antithyroglobulin antibody; MDA: malondialdehyde; TAC: total antioxidant capacity; SOD: superoxide dismutase.

**Table 4:** Correlation analysis between the oxidative stress parameters and thyroid autoantibodies titers in pretreatment AIT patients and healthy controls.

Group	Variables	TAC		SOD		MDA	
		*r*	*P*	*r*	*P*	*r*	*P*
Controls	TgAb	−0.103	0.392	−0.367	0.096	−0.190	0.289
	TPOAb	0.112	0.829	0.490	0.195	−0.365	0.102

Patients	TgAb	−0.268	0.039^*∗*^	−0.237	0.102	0.429	0.041^*∗*^
	TPOAb	−0.463	0.008^*∗*^	−0.385	0.074	0.587	0.023^*∗*^

## Data Availability

The data used to support the findings of this study are available from the corresponding authors upon request.
